# Shorter axon initial segments do not cause repetitive firing impairments in the adult presymptomatic G127X SOD-1 Amyotrophic Lateral Sclerosis mouse

**DOI:** 10.1038/s41598-019-57314-w

**Published:** 2020-01-28

**Authors:** V. S. Bonnevie, K. P. Dimintiyanova, A. Hedegaard, J. Lehnhoff, L. Grøndahl, M. Moldovan, C. F. Meehan

**Affiliations:** Department of Neuroscience, University of Copenhagen, Panum Institute, Blegdamsvej 3, DK-2200 Copenhagen N, Denmark

**Keywords:** Amyotrophic lateral sclerosis, Ion channels in the nervous system

## Abstract

Increases in axonal sodium currents in peripheral nerves are some of the earliest excitability changes observed in Amyotrophic Lateral Sclerosis (ALS) patients. Nothing is known, however, about axonal sodium channels more proximally, particularly at the action potential initiating region - the axon initial segment (AIS). Immunohistochemistry for Nav1.6 sodium channels was used to investigate parameters of AISs of spinal motoneurones in the G127X SOD1 mouse model of ALS in adult mice at presymptomatic time points (~190 days old). I*n vivo* intracellular recordings from lumbar spinal motoneurones were used to determine the consequences of any AIS changes. AISs of both alpha and gamma motoneurones were found to be significantly shorter (by 6.6% and 11.8% respectively) in G127X mice as well as being wider by 9.8% (alpha motoneurones). Measurements from 20–23 day old mice confirmed that this represented a change during adulthood. Intracellular recordings from motoneurones in presymptomatic adult mice, however, revealed no differences in individual action potentials or the cells ability to initiate repetitive action potentials. To conclude, despite changes in AIS geometry, no evidence was found for reduced excitability within the functional working range of firing frequencies of motoneurones in this model of ALS.

## Introduction

Amyotrophic Lateral Sclerosis (ALS) is a fatal neurodegenerative disease which preferentially affects motoneurones in the brain and spinal cord. Although a number of different mutations have been found to account for a small proportion of cases, the underlying pathophysiology of the disease is not fully understood and there is currently no cure. Riluzole, the only established treatment, has only modest effects on survival time^1–5^ and blocks glutamatergic neurotransmission and Na^+^ and Ca^2+^ channels, indicating that excitability plays a role in the degenerative process in ALS.

Threshold tracking techniques have suggested a hyperexcitability of motoneurone axons in ALS patients consisting of an increase in sodium currents occurring with a decrease in potassium currents^6–14^. The magnitude of the ion current changes appears to correlate with disease progression^15^, with excitability changes occurring before detectable axon loss^7,15^. In humans, the cell body and proximal axons of motoneurones, however, are largely inaccessible, preventing direct investigations of sodium and potassium channel activity centrally in ALS patients. This has therefore been investigated *in vitro* in motoneurones cultured from induced pluripotent stem cells (iPSCs) from ALS patients with genetic forms of the disease including superoxide dismutase 1 (SOD1), C9orf72 repeat expansions, TAR DNA binding proteins (TARDBP) and fused-in-sarcoma (FUS) mutations^16–19^. Increased action potential firing is observed at early stages after plating^16,19^ due to reductions in delayed rectifier potassium currents^19^ and increases in peak sodium currents^16^. At later time points post-plating however, motoneurones exhibit deficits in repetitive firing^16,18^ due to elevated potassium currents and attenuated sodium currents^17^ or attenuations in both^16,18^. Motoneurones derived from human iPSCs have been shown, however, to be more similar to fetal spinal tissue than to adult spinal motoneurones^20^. Furthermore, experiments exposing wild type cultured rodent motoneurones to astrocytes harbouring SOD1 or TDP43 mutations suggest that local astrocytes may be necessary to drive excitotoxic increases in persistent inward sodium currents^21,22^.

*In vitro* investigations of cultured motoneurones from the transgenic G93A SOD1 mouse model of the disease have shown increases in persistent sodium currents^23,24^ with sodium channels displaying a faster recovery from fast inactivation than controls^25^. Recordings from neonatal spinal slice preparations from the same mice have also confirmed an early increase in both Na^+^ and Ca^2+^ persistent inward currents (PICs)^26^. ALS however, is an adult onset disorder, therefore it is crucial to establish whether the motoneurones exhibit abnormal excitability in adulthood. Furthermore, it is important to determine this *in vivo*.

Our previous *in vivo* investigations in the adult G127X SOD1 mouse model of ALS have confirmed a presymptomatic increase in Na^+^ current in distal peripheral motor axons^27^ and a disruption of axonal potassium channels related to a breakdown of nodal organisation in the ventral roots of these mice, assumed to be indicative of axonal degeneration^28^. Electrophysiological recordings have, however, provided no evidence for a decrease of potassium channels centrally, even at the symptomatic stage^28^. In the current experiments we now focus on central sodium channels in the same transgenic mouse model at pre-symptomatic adult time points with a particular focus on the axon initial segment (AIS). We hypothesized the AIS to be affected for a number of reasons. First, activity dependent plasticity of the AIS *in vitro* has been shown to be driven by L-type calcium channel activity^29,30^ and we have previously shown an increase in PICs mediated by these channels in this model in pre-symptomatic adults^31^. Second, reductions in AIS length have been shown to impair repetitive firing^32^ and recordings in the adult G93A SOD1 mouse model of ALS have suggested impairments in repetitive firing in the motoneurones^33^. Finally, anatomical investigations of post-mortem ALS patients have also shown a swelling of proximal motoneurone axons (including AISs) compared to controls^34,35^. In the present experiments immunohistochemistry was used to label Nav1.6 sodium channels at AISs of spinal motoneurones in adult presymptomatic G127X SOD1 mice combined with *in vivo* intracellular recording to identify the functional significance of any changes.

## Results

### Alpha motoneurone axon initial segments are shorter and wider in adult G127X mice

**Figure 1:**
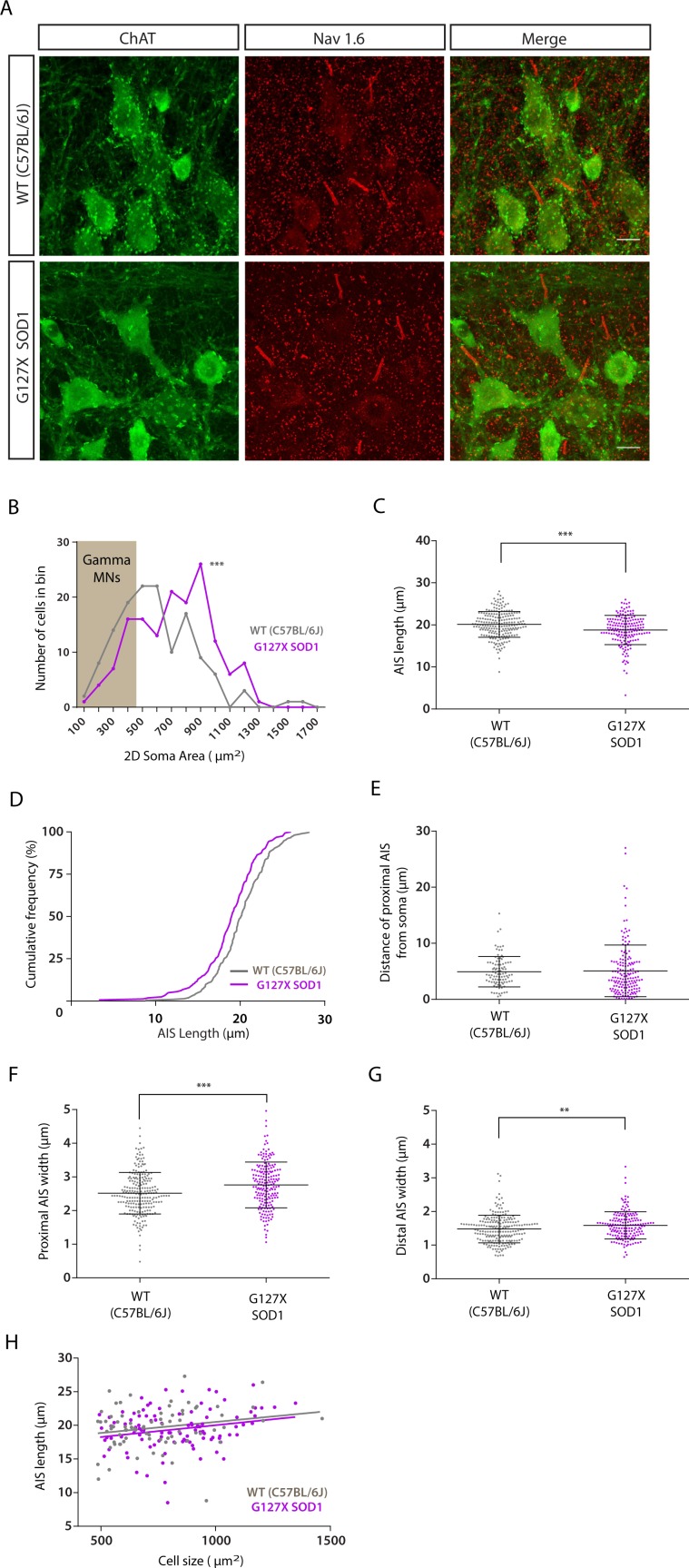
AIS parameters of alpha motoneurones in WT (grey) and G127X (magenta) mice. (**A**) Maximum intensity projections from confocal stacks of immunohistochemical labelling for Nav 1.6 (red) and ChAT (as a marker of motoneurones, green) in WT and G127X mice. Scale bar 20 µm. (**B**) Frequency distribution of 2D soma area of motoneurones in WT and G127X mice grouped in 100µm^2^ bins. Gamma motoneurones can be excluded by their size (grey box <485 µm^2^). From this it can be seen that the alpha motoneurones of G127X mice as a population are significantly larger than those of the WT motoneurones. (**C**) Scatter dot plot of AIS length of motoneurones in WT and G127X mice. AISs of motoneurones in G127X mice are significantly shorter than in WT. (**D**) Cumulative frequency distribution of AIS length for WT and G127X showing that the entire distribution has shifted towards shorter AIS lengths in the G127X mice. (**E**) Scatter dot plot showing the distance of the proximal AIS from the soma of motoneurones in WT and G127X mice. From this, no significant difference can be seen. (**F**,**G**) Scatter dot plots showing the width of the proximal (**F**) and distal (**G**) AIS of motoneurones in WT and G127X mice. From this it can be observed that both proximal and distal AISs of the G127X mice are wider than WT (but the effect is more pronounced for proximal AISs). (**H**) Regression line for AIS length by soma size for WT and G127X, where it can be seen that smaller motoneurones tend to have shorter initial segments. Bars on scatter dot plots show means with SD and data points represent individual cells.

Axon initial segments were immunohistochemically labelled using antibodies against Nav1.6 (the main sodium channel subtype found at the AIS) and antibodies against choline acetyltransferase (ChAT) to label spinal motoneurones in the ventral horn of the lumbar spinal cord in adult (~193 day old) presymptomatic ALS mice and aged-matched wild type (WT) mice (more details of all mice used can be found in Table 1). Examples of motoneurone AISs in WT and G127X mice are shown in Fig. 1A. Only AISs connected to ChAT immunoreactive motoneurones were measured. For those motoneurones with the entire soma contained within the 50 µm thick tissue section, the 2D area was calculated using the maximum and minimum diameters. AISs belonging to soma with areas smaller than 485 µm^2^ (gamma motoneurone) were taken out for separate analysis and all remaining cells were assumed to be alpha motoneurones (Fig. 1B). The results are summarised in Table 2 and the spread of individual data points can be seen in the scatter plots shown in Fig. 1C,E–H. AISs of alpha motoneurones were slightly (6.6%) but significantly shorter in the G127X mice (Fig. 1C). When this data is displayed as cumulative frequency fractions it can be seen that this difference is not due to a selective loss at one end of the distribution but an overall shift of all values in the G127X mice towards shorter lengths (Fig. 1D). The mean distance of the AIS from the soma was not statistically significant, (Fig. 1E) but AISs were significantly wider in G127X mice at both their proximal and distal ends (by 9.8% and 7.2% respectively, Fig. 1F,G).

**Table 1:** Details of the adult mice used for the different parts of the investigation.

Investigation	Group	N (mice)	Male/Female	Mean Age (and range) in days
Anatomy: Adult AIS measuring*	WT	6	2/4	193 (182–210)
	G127X	6	5/1	192 (187–202)
Anatomy: Cell counting	WT	4	4/0	205 (193–240)
	G127X	5	5/0	204 (170–222)
Electrophysiology**	WT	6	0/6	195 (182–210)
	G127X	11	4/7	214 (187–230)

*3 of the G127X mice and 4 of the WT mice were used for both electrophysiology and AIS labelling experiments.

**Mice used for the electrophysiological recordings were also used for experiments investigating axonal potassium channels (Maglemose *et al*. 2017).

**Table 2:** Parameters of axon initial segments of alpha motoneurones in adult WT and G127X mice.

	Length (μm)	Distance from soma (µm)	Proximal width (µm)	Distal width (µm)	2D Soma size (µm^2^)
WT	20.12 (3.066) n = 210	4.928 (2.699) n = 94	2.514 (0.6165) n = 224	1.48 (0.4114) n = 212	743.4 (216.4) n = 82
G127X	18.79 (3.465) n = 168	5.087(4.602) n = 159	2.760 (0.6774) n = 191	1.586 (0.4034) n = 167	823 (196) n = 123
Significance	**P = 0.0005**	P = 0.1613	**P = 0.0001**	**P = 0.0080**	**P = 0.0010**
Statistical test	Mann Whitney	Mann Whitney	Unpaired t test	Mann Whitney	Mann Whitney
Statistical value	U = 13951	U = 6685	t = 3.88 (df 412)	U = 14894	U = 3677

Means are given with SD in brackets.

The soma of the alpha motoneurones were significantly (10.7%) larger in the G127X mice than WT. To determine if this was, in anyway, related to the changes in AIS parameters, linear regressions were performed between soma size and AIS length, distal width and distance from soma respectively. Surprisingly, there was no significant relationship between soma size and any of the AIS parameters for both groups except one feature. In the G127X mice there was a significant relationship between soma size and AIS length with smaller motoneurones having shorter AISs (Fig. 1H). This suggests that the difference in AIS length between WT and G127X mice was not simply due to the motoneurones being larger in the G127X group. Linear regression also showed that in the WT mice longer AISs were usually further from the cell body (data not shown). This relationship was absent in the G127X mice.

### Gamma motoneurone AISs are shorter and thinner in adult G127X mice

**Figure 2:**
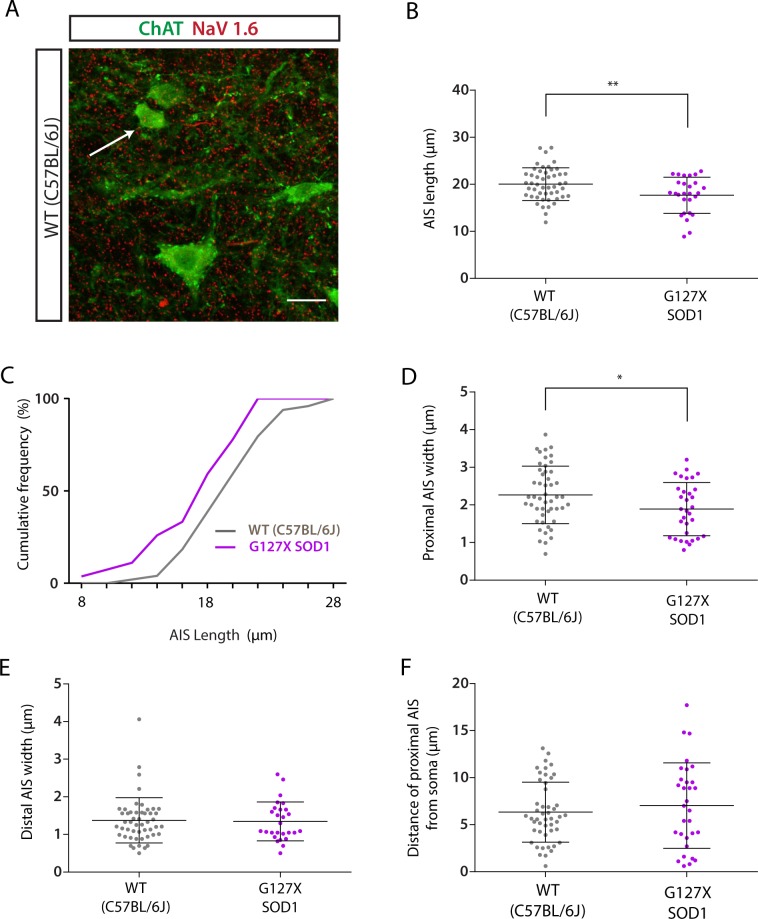
AIS parameters of gamma motoneurones in WT (grey) and G127X (magenta) mice. (**A**) Maximum intensity projections from confocal stacks of immunohistochemical labelling for Nav 1.6 (red) and ChAT (as a marker of motoneurones, green) in a WT mouse. The white arrow points to a gamma motoneurone identified by size and a lack of C-boutons. Scale bar 20 µm. (**B**) Scatter dot plot of AIS lengths in WT and G127X mice showing that AISs of gamma motoneurones in the G127X mice are also significantly shorter (P = 0.0163) than in WT. (**C**) Cumulative frequency distribution of AIS length for WT and G127X showing that the entire distribution has been shifted towards shorter AIS lengths in the G127X mice. (**D**,**E**) Scatter dot plot of the width of proximal (**D**) and distal (**E**) AISs in WT and G127X mice showing that AISs of the G127X mice are thinner proximally but not distally. (**F**) Scatter dot plot showing the distance of the proximal AIS from the soma of gamma motoneurones in WT and G127X mice. From this a trend can be seen for AISs of the G127X mice to be slightly further from the soma compared to the WT. Bars on scatter dot plots show means with SD and data points represent individual cells.

Differences were also observed for AISs of gamma motoneurones (Table 3, Fig. 2A–D) although a smaller sample was obtained. AISs from G127X mice were on average 11.8% shorter than WT (Fig. 2B). Again, when the length data is displayed as cumulative frequency fractions it can be seen that this difference is not due to a selective loss at one end of the distribution but an overall shift of all values in the G127X mice towards shorter lengths (Fig. 2C). AISs from G127X mice were also 16.6% thinner proximally (Fig. 2D), but no significant differences were observed for either distal width (Fig. 2E) or distance from soma (Fig. 2F). Given that size was used as an identifying feature for gamma motoneurones, size was not investigated further.

**Table 3:** Parameters of axon initial segments of gamma motoneurones in adult WT and G127X mice.

	Length (µm)	Distance from soma (µm)	Proximal width (µm)	Distal Width (µm)
WT	20.04 (3.471) n = 49	6.334 (3.187) n = 48	2.265 (0.7645) n = 49	1.373 (0.602) n = 50
G127X	17.67 (3.84) n = 27	7.035 (4.545) n = 31	1.888 (0.7044) n = 32	1.347 (0.5174) n = 27
Significance	**P = 0.0077**	P = 0.4222	**P = 0.0398**	P = 0.8882
Statistical test	Unpaired t test	Unpaired t test	Mann Whitney	Mann Whitney
Statistical value	t = 2.74 (df = 74)	t = 0.8069 (df = 77)	U = 571.5	U = 661.5

Means are given with standard deviations in brackets.

### Minimal motoneurone loss is observed at this age in the adult G127X mice

**Figure 3:**
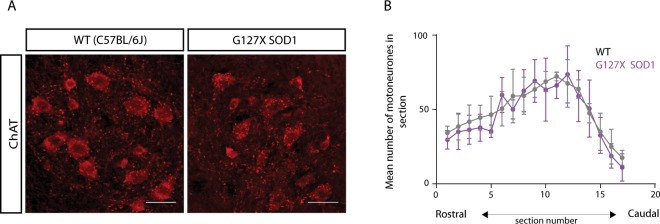
Motoneurone cell loss in the adult G127X mouse is minimal. (**A**) Maximum intensity projections from confocal stacks showing examples of ChAT immunoreactive motoneurones in the ventral horn from a WT (left) and a G127X (right) mouse (scale bar is 50 µm). (**B**) Graph showing the mean number of motoneurones at each level (sections taken every 500 µm on the x-axis) for WT (grey, 4 mice) and G127X (magenta, 5 mice). The distributions for the individual mice have been aligned using the intermediolateral cell column (L2) and the start of the parasympathetic preganglionic cell columns (S1) and the maximum peak as guidance. Scatter plots show mean and error bars, SD.

To determine whether changes in AISs of both alpha and gamma motoneurones were due to a selective cell loss we quantified cell loss at this age in 5 presymptomatic G127X and 4 WT mice of a similar age using ChAT labelling (Fig. 3A). The number of motoneurones was counted in every 10^th^ transverse section from the lumbar spinal cord and aligned as described in the methods. The mean number of motoneurones at each level for both groups is shown in Fig. 3B. From this, no clear differences could be observed confirming that there is not an obvious loss of motoneurones at this age in G127X mice. The mean number of motoneurones per section was 49.12 (WT) and 46.53 (G127X) which was not significantly different (P = 0.2857, Mann Whitney, U = 5).

### Shorter AISs of alpha motoneurones represent a developmental change in adult G127X mice, a feature not observed in WT mice

**Figure 4:**
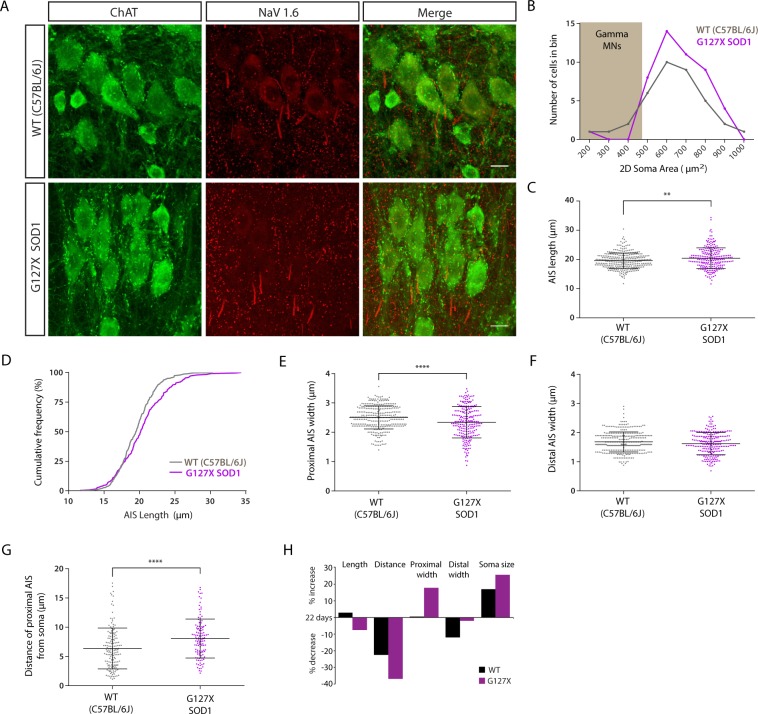
AIS parameters of alpha motoneurones in ~22 day old WT (grey) and G127X (magenta) mice (**A**) Maximum intensity projections from confocal stacks of immunohistochemical labelling for Nav 1.6 (red) and ChAT (green) in ~22 day old WT and G127X mice. Scale bar 20 µm. (**B**) Frequency distribution for 2D motoneurone soma area for WT and G127X mice (grouped in 100 µm bins) showing no significant difference between the groups (gamma motoneurones can be excluded by their size (grey box <485 µm^2^). (**C**) Scatter dot plot showing the AIS length in WT and G127X mice showing that, at this age, the AISs of motoneurones in G127X mice are significantly longer than in WT. (**D**) Cumulative frequency distribution for AIS length showing that there are a greater number of longer AISs in the G127X mice than in WT. (**E**,**F**) Scatter dot plot of the width of proximal (**E**) and distal (**F**) AISs in WT and G127X mice. From this it can be seen that AISs of the G127X mice are thinner proximally but not distally. (**G**) Scatter dot plot showing the distance of the AIS from the soma in ~22 day old WT and G127X mice. From this it can be observed that at this age the AIS of motoneurones in the G127X mice are further away from the soma than in WT. (**H**) Histogram showing how the different parameters of the AIS in the adult mice have changed relative to the same parameters at ~22 day (zero on Y axis). From this it can be concluded that for most parameters, the magnitude of the change is different but in the same direction except length which increases slightly in WT mice and decreases in G127X mice. Bars on scatter plots show means with SD.

To investigate whether the changes that we observed in the AISs of G127X mice represent plastic changes during adulthood or exist from birth we measured AISs in ~22-day old WT and G127X mice. Examples are shown in Fig. 4A, AIS parameters are summarised in Table 4 and the spread of individual data points can be seen in the scatter plots shown in Fig. 4. AISs were actually slightly longer (by 3.9%) in the 22-day old G127X mice compared with WT (Fig. 4C). Displayed as cumulative frequency fractions, the data suggests that in the G127X population there is a greater number of longer AISs (Fig. 4D). AISs in the G127X mice were also significantly thinner proximally (by 6.4%, Fig. 4E) but not distally (Fig. 4F) and further from the soma (Fig. 4G) compared to WT. So, although the AISs of the motoneurones are different from WT at a young age in the G127X mice, these differences are not in the same direction as observed in adults at ages close to but before symptom onset.

**Table 4:** Parameter of axon initial segments of alpha motoneurones in 22-day old WT and G127X.

	Length (µm)	Distance from soma (µm)	Proximal width (µm)	Distal width (µm)	2D Soma size (µm^2^)
WT_(22 days)_	19.57 (2.62) n = 319	6.37 (3.49) n = 154	2.5 (0.39) n = 319	1.68 (0.34) n = 319	635.6 (165.1) n = 37
G127X _(22 days)_	20.41 (3.57) n = 241	8.08 (3.33) n = 119	2.34 (0.53) n = 239	1.62 (0.38) n = 240	654.5 (130.4) n = 47
Significance	**P = 0.0068**	**P = <0.0001**	**P < 0.0001**	P = 0.0649	P = 0.5588
Statistical test	Mann Whitney	Mann Whitney	Unpaired t test	Unpaired t test	Unpaired t test
Statistical value	U = 33305	U = 6304	t = 4.248 (df = 556)	t = 1.850 (df = 561)	t = 0.587 (df = 82)

Means are given with standard deviations in brackets.

To evaluate how the changes in the AIS parameters between ~22 and ~193 days on G127X mice represent normal, abnormal or enhanced development we first compared the parameters between WT mice at ~22 days and the WT adults. How the different parameters of the AIS in the adult WT and G127X mice have changed relative to the same parameters at ~22 day (zero on Y axis) is illustrated in Fig. 4H. From this it can be seen that for most parameters the magnitude of the change is different between WT and G127X but in the same direction except length. In adult WT mice, as the mice mature, a small increase (2.8%) in the length of the AIS is seen (P = 0.008) and AISs move closer to the soma (by 22.6%, P < 0.015) and are thinner at their distal ends (by 11.9%, P = 0.0049) but with no changes to the width of the proximal AIS. In the adult G127X mice the AISs also showed a trend to become thinner distally (2.1%, P = 0.0904) and moved significantly closer to the soma, in fact, this was much more pronounced (37.1% closer, P < 0.0001). This most likely explained why AISs become wider proximally (by 17.9%, P < 0.0001) as they come closer to the axon hillock which is a wider structure. The main difference, however, was that, unlike in WT mice, the AISs in the G127X mice actually became shorter with age (by 7.6%, P = 0.0004) instead of longer. Therefore, this decrease in AIS length in the 190 day old G127X mice is in contrast to the normal changes seen during development.

### Electrophysiological results

To determine whether the changes in AIS parameters observed in the adult mice can have a functional consequence, we investigated excitability parameters related to sodium channels and action potential generation in the adult WT and G127X mice using *in vivo* intracellular recording with sharp microelectrodes. Intracellular recording techniques *in vivo* are generally biased towards recording from alpha motoneurones rather than gamma due to their bigger cell size. We therefore assume our recordings to represent alpha motoneurones. All motoneurones used for analysis were antidromically identified by stimulation of the sciatic nerve.

### Action potential height and rate of rise is not different in the G127X mice

**Figure 5:**
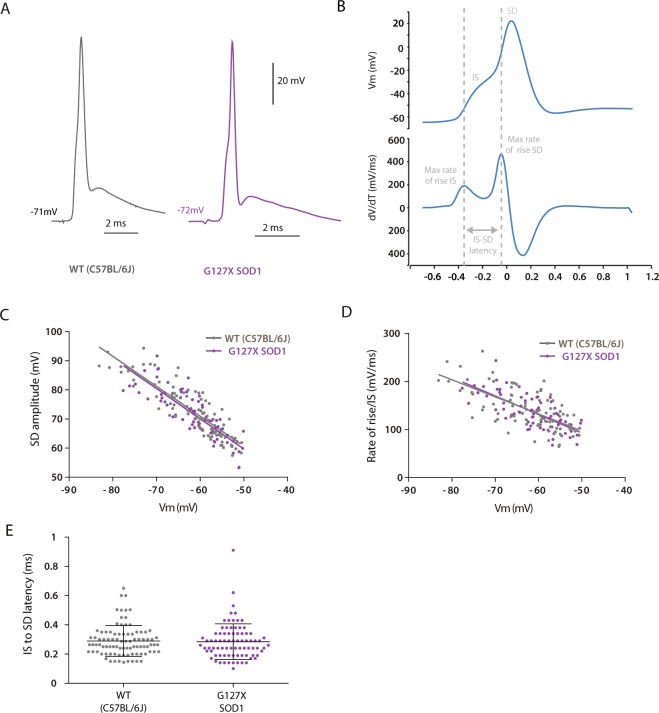
Features of antidromic action potentials are unaltered in WT (grey) and G127X (magenta) mice. The features of antidromic action potentials were recorded in 6 WT and 8 G127X mice (where sampling frequency was optimal to allow reliable analysis). (**A**) Examples of averages (using ≥10 spikes) of antidromic action potentials in motoneurones from WT and G127X mice. (**B**) An example of an antidromic action potential (upper trace) from a WT mouse with its first derivative (lower trace) showing how the maximum rate of rise for the IS and SD components of the action potential were obtained and how the IS-SD latency was measured. (**C**) Regression lines for amplitude of the soma-dendritic (SD) component of the antidromic action potential with respect to membrane potential (Vm) for motoneurones from WT and G127X mice. No significant differences between the regression lines can be seen. (**D**) Regression lines for rate of rise of initial segment (IS) component of the antidromic action potential with respect to membrane potential (Vm), showing no significant differences between WT and G127X mice. (**E**) Scatter dot plot showing individual data points for IS-SD latency for motoneurones (measured as the time between the maximum rate of rise of the IS and the SD components of the antidromic action potential) showing no significant differences between WT and G127X mice (bars show means with SD).

Antidromic action potentials were recorded from 89 motoneurones in WT mice and 71 in G127X mice, examples of which are shown in Fig. 5A. The spike height of the somato-dendritic (SD) spike was measured from baseline to peak, however, the amplitude of the initial segment (IS) action potential cannot be measured directly as it is masked by the SD action potential. The average of the action potential was therefore differentiated to obtain the maximum rate of rise of the IS component (Fig. 5B, lower trace). Linear regression confirmed that the amplitude of the SD action potential correlated with baseline membrane potential, Vm (WT: R^2^ = 0.7788, P < 0.0001; G127X: R^2^ = 0.7014, P < 0.0001, Fig. 5C). No significant differences were found with respect to both the slopes (P = 0.9186) and intercepts (P = 0.2156) of the regression lines between WT and G127X mice.

Linear regression also confirmed that the maximum rate of rise of the IS component of the antidromic action potential correlated with Vm (WT: R^2^ = 0.4488, P < 0.0001; G127X: R^2^ = 0.4221, both P < 0.0001, Fig. 5D). Again, no significant differences were found between the slopes (P = 0.6294) and intercepts (and P = 0.6381) of the 2 regression lines for action potentials from WT and G127X mice. Finally, the IS-SD latency was calculated as the latency between the maximum rate of rise of the IS and SD components of the antidromic action potential (Fig. 5B). The mean IS-SD latency for WT was 0.29 ms (SD 0.105) and for G127X it was 0.2812 ms (SD 0.131) which was not significantly different between WT and G127X mice (P =  0.3959 Mann Whitney U = 3332, Fig. 5E).

### Recovery from inactivation is reduced at the soma but not AISs of G127X mice

**Figure 6:**
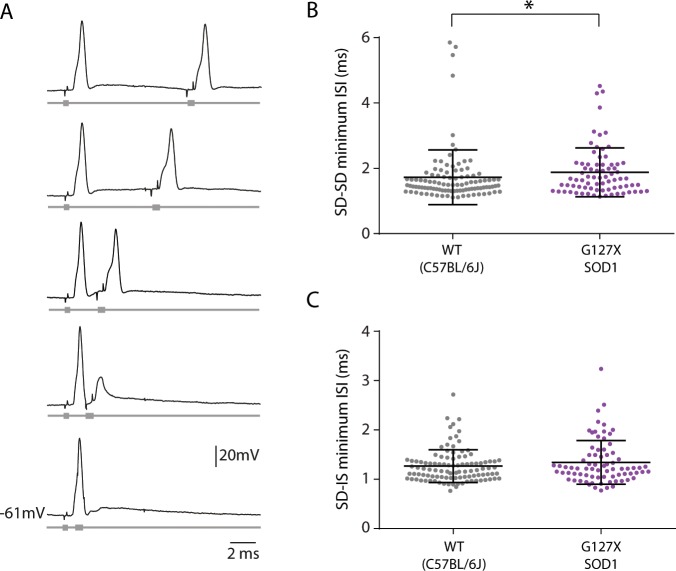
Recovery from inactivation is reduced at the Soma but not at the AIS of G127X mice. Recovery from inactivation was tested in 6 WT and 9 G127X mice. (**A**) Example showing the protocol used to test the recovery from inactivation of the antidromic action potentials. Black traces show intracellular recordings and grey traces beneath show the corresponding stimulation times. Two stimulations were given in close succession and the interval between the two action potentials was decreased until the second action potential failed. The minimum inter-spike interval for the two SD action potentials (ISI_min_ SD-SD) was measured from peak to peak of the last successful action potential before failure of the second spike (e.g. the third pair of traces). In the 4th pair of traces, it can be seen that the SD component of the action potential has failed but the IS component persists. In the bottom traces the IS component has failed, too. The point just before the IS action potential fails is referred to as ISI_min_ SD-IS and was measured from the peak of the SD spike to the peak of the IS spike. (**B**) Scatter dot plot showing individual data points for the minimum interspike interval for soma-dendritic antidromic action potentials (ISI_min_ SD-SD, e.g. the third pair of traces on A) for motoneurones from the WT (grey) and G127X (magenta) mice. From this it can be observed that SD spike failure occurs at significantly longer inter-spike intervals in G127X mice (bars show means with SD). (**C**) Scatter dot plot showing the individual data points for the minimum interspike interval for initial segment action potentials (ISI_min_ SD-IS, e.g. the fourth pair of traces on A) for motoneurones from WT (grey) and G127X (magenta) mice (bars show means with SD). No significant differences can be seen between the two groups.

To test how quickly the antidromic spike could recover from inactivation, a second stimulation was given in close succession and the interval between the two action potentials decreased until the second action potential failed (Fig. 6A). The last measurement of both spikes, just before the second spike fails, is referred to as the minimum inter-spike interval for the two SD action potentials (ISI_min_ SD-SD), measured from peak to peak of the two action potentials. Often, when the SD component of the second spike failed, the IS spike persisted (Fig. 6A). The point just before the second IS action potential fails is referred to as ISI_min_ SD-IS. Recovery from inactivation was tested in a total of 103 motoneurones in WT and 76 motoneurones in G127X mice.

The ISI_min_ SD-SD was significantly longer in G127X mice (means (+SD) WT: 1.724 ms (0.838), G127X. 1.876 ms (0.7466), Mann Whitney U = 3224, P = 0.0438, Fig. 6B), suggesting that action potentials at the soma have a longer recovery time from inactivation. The ISI_min_ SD-IS, however, was not significantly different between WT and G127X mice (means (+SD) WT: 1.267 ms (0.333), G127X. 1.342 ms (0.4434), Mann Whitney U = 3689, P = 0.5119, Fig. 6C), suggesting that at the AIS, the recovery from inactivation is unchanged in G127X mice.

### Motoneurones in the G127X mouse retain the ability to fire repetitively in response to intracellular current injection

**Figure 7:**
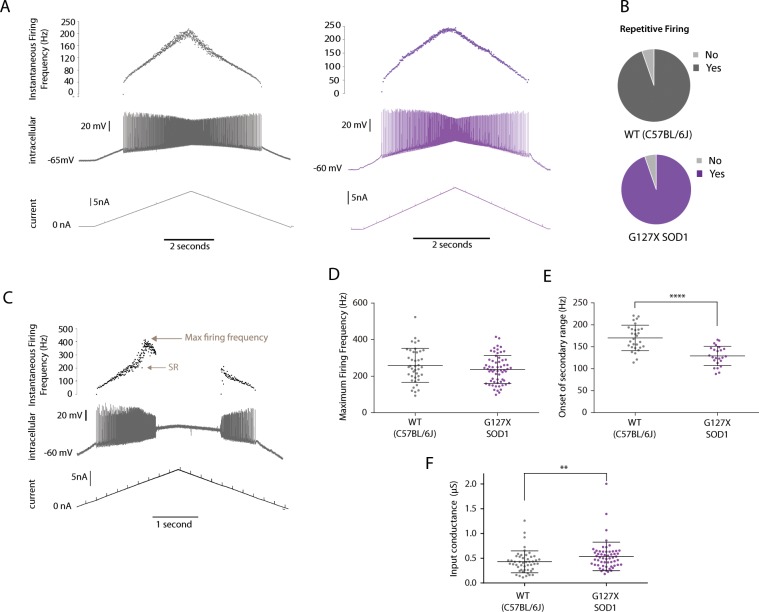
Repetitive firing in response to intracellular current injection is unaltered in G127X (magenta) compared to WT (grey) mice. The ability to fire repetitively in response to triangular current injection to the soma was tested in 6 WT and 11 G127X mice. (**A**) Examples of repetitive firing recorded intracellularly (middle traces) in motoneurones from a WT and a G127X mouse in response to intracellular injection of ramps of ascending and descending intensity of current (bottom trace). The instantaneous firing frequency is shown on the top trace. (**B**) The proportions of motoneurones in WT and G127X mice responding to the current injection with repetitive firing, showing no differences between the groups. (**C**) Example of a motoneurone from a WT mouse where the ascending phase of current injection was maintained until the full action potential failed. From this, the maximum firing frequency could be obtained. An inflection in the firing frequency (upper trace) indicates the onset of the secondary range (SR) of firing representing the onset of Ca^2+^ persistent inward currents. (**D**) Scatter dot plot showing individual data points for the maximum firing frequency of motoneurones from WT and G127X mice. No significant differences were seen between WT and G127X mice. (**E**) Scatter dot plot showing individual data points for the firing frequency at onset of secondary range in motoneurones from WT and G127X mice. Here is can be seen that the secondary range onsets significantly earlier in motoneurones from G127X mice. (**F**) Scatter dot plot showing individual data points for the input conductance of motoneurones from WT and G127X mice estimated from hyperpolarizing currents pulses. Here it can be seen that the mean inputs conductance is significantly higher in the G127X mice. Bars on scatter plots show means with SD.

To test the ability of the motoneurones to fire repetitively in response to central inputs, intracellular current injection was used to mimic synaptic input. Triangular ramps of current were injected through the microelectrode and the cell’s ability to fire repetitively determined. Examples from both WT and G127X mice are shown in Fig. 7A. Before the cell was tested, it was first confirmed that electrode capacitance was correctly compensated (using the capacitance compensation on the Axoclamp 2b amplifier) and that the electrode was passing the current, which is important given the high resistance electrodes used for these studies. The membrane potential immediately prior to current injection was confirmed to be more hyperpolarised than −50 mV as, in our experience, more depolarized membrane potentials rarely exhibit repetitive firing. Triangular current ramps evoked repetitive firing in 94.6% (53/56) motoneurones in WT mice and 94.7% (71/75) motoneurones in G127X mice (Fig. 7B). It is worth noting that the motoneurones failing to fire repetitively had membrane potentials relatively close to −50 mV. Therefore, there was no significant impairment in repetitive firing in response to intracellular current injection in G127X mice compared to WT. In 90 motoneurones (28 WT and 62 G127X) the current injection was taken to the level where repetitive firing of full spikes failed, in order to determine the maximum firing frequencies in response to the ramp current injection (Fig. 7C). This was not significantly different between the two groups (P = 0.7550, t-test t = 0.313, mean (+SD): WT 242.3 Hz (94.17), G127X 236.3 Hz (76.37), Fig. 7D). Consistent with our previous study^31^, an inflection in the current-frequency slopes corresponding to the onset of the secondary range^36^, due to the onset of calcium PICs, was frequently observed, (Fig. 7C). When observed, this occurred at significantly lower firing frequencies in the G127X mice (P = <0.0001, t-test t = 5.694, means (+SD), WT:  170 Hz (28.9), 29 cells, G127X: 129 Hz (22.2), 24 cells, Fig. 7E). In the majority of the cells in which repetitive firing was tested (49 WT and 57 G127X), short hyperpolarizing current pulses (also in DCC mode) were used to estimate the input conductance to test if this may compensate for the increased PICs. This was found to be significantly higher in G127X motoneurones (P = 0.0097, Mann-Whitney, U = 990, means (+SD), WT: 0.427 µS (0.222), G127X: 0.537 µS (0.2872), Fig. 7F).

## Discussion

Subtle differences were found with respect to the geometric AIS parameters in the presymptomatic G127X SOD1 mouse model of ALS. Despite these changes, no significant differences were observed with respect to features of individual action potentials between the two groups except a slight reduction in the recovery time for 2 successive somatic action potentials at very high firing frequencies. None of the observed changes impaired the ability of the G127X motoneurones to fire repetitively or limit their maximum firing frequencies.

### Plasticity of the AIS

The AISs of alpha motoneurones in adult G127X mice were both significantly shorter and wider than in WT mice. Our measurements in ~22 day old mice confirmed that this was a plastic change in adulthood not observed in WT mice. Despite shorter AISs, no functional changes were observed with respect to the rate of rise of the AIS action potential, suggesting no overall change in the number of Na^+^ channels. Using the mean distal AIS width to calculate the AIS diameter together with the mean AIS length we can calculate the mean surface area of the AIS for the 2 groups. These are remarkably similar (WT: 96.99 μm^2^ and G127X 97.57 μm^2^). If we are to assume that the overall density of ion channels does not change (which is technically challenging to check with a highly fixation-dependent antibody), then this would mean that there is a similar surface area of sodium channels in both groups which could explain why no differences were observed with respect to the rate of rise of the AIS action potentials between the two groups.

We have previously observed from a larger sample of rat motoneurones that AIS width correlates with soma size, with larger motoneurones having wider AISs^37^. Furthermore, wider AISs were usually shorter and located more proximal to the somas. The changes in AIS parameters that we have observed in the current experiments could therefore be explained by a compensatory response to the increase in soma size in the G127X mice.

One concern is that the unequal distribution of males and females in each group may have influenced the results, however, we have previously found in another study that the AIS parameters of mice motoneurones are not significantly different between adult male and female C57BL mice^37^, although differences in cell size could be potentially explained in this way. In another, different SOD1 model, the G93A mouse, an increase in cell size has also been observed of a similar magnitude (~8%)^38^ to what we observed in the G127X mouse (~12%).

Gamma motoneurones also showed a reduction in the length of the AIS but with a reduction in AIS width and no changes in the distance from the soma suggesting a decreased excitability of these neurones. Being much smaller than alpha motoneurones, gamma motoneurones possess a gamma bias, producing a constant drive to the extrafusal muscle fibres. A decrease in gamma motoneurone excitability would therefore be expected to reduce the sensitivity of the muscle spindles to changes in muscle length. Muscle stretch usually results in high frequency trains of action potentials in Ia afferents causing Excitatory Post Synaptic Potentials (EPSPs) in spinal motoneurones which are normally reduced in amplitude at these high frequencies due to post activation depression. We have previously shown that post activation depression of the Ia EPSP is reduced in the G127X mouse at a similar presymptomatic age to the current study^39^. Such a reduction in depression equates to a subtle increase in the amplitude of Ia EPSPs which may compensate for reduction in the sensitivity of the muscle spindles potentially due to reduced gamma motoneurone excitability. Reductions in post activation depression is commonly observed in disorders characterised by the feature of spasticity, it is therefore of interest to explore if the excitability of gamma motoneurones increases at symptom onset in this same model.

### Changes in AIS parameters of alpha motoneurones are consistent with changes in proximal motor axons in post-mortem ALS patients

Although the AISs of motoneurones of human ALS patients have not been measured using precise molecular markers of the AIS, electron microscopy (EM) has been used to examine the proximal axons of ventral horn neurones from post-mortem patients with ALS. These have shown a swelling of distal AISs (by around 21% in normal looking axons and 117% in swollen axons) and a decrease in the combined length of the AIS and axon hillock (measured as the initial portion of the axon hillock to the point at which myelination started) by 4% in normal looking axons and 7.3% in swollen looking axons^35^. Our results confirm that similar changes also occur in this animal model of the disease. Furthermore, the fact that we have observed this in pre-symptomatic mice suggests that this is an early event in the disease process. The EM studies with the human tissue show the proximal axons to contain abnormal accumulations of neurofilaments, lysosomes, vesicles and mitochondria^35,40^ suggesting that deficits in axon transport may underlie the swelling of the AISs. Similar accumulations of neurofilaments and mitochondria have also been observed in the proximal axons of motoneurones in the G93A SOD1 mouse model^41^ suggesting that this could equivalently explain the changes in AIS diameter in adulthood in the G127X SOD1 model.

### Repetitive firing is not impaired in the G127X mouse

Our electrophysiological recordings confirmed that the changes in AIS parameters did not impair the neurones ability to fire repetitive action potentials. This is in contrast to data recently published in adult G93A SOD1 mice^33^ where a larger number of cells were unable to fire repetitively compared with WT. There are a number of possible reasons for this difference.

One major difference between the two models is in the timing of symptom onset and disease progression. In the G93A mouse strain, mice start to develop paralysis by 90 days of age followed by a disease course lasting approximately 30 days^33^. The G127X mouse, by contrast, does not develop clear symptoms until approximately 250 days of age, followed by a rapid disease progression of around 10 days^27^. It is therefore possible that the adult pre-symptomatic time points used in the two different studies (G93A: 38 to 82 days and G127X: 213 days) represent different time points in the disease progression. As cells are beginning to degenerate much earlier in the G93A mice, it is possible that in the experiments of Delestrée *et al*. the increase in non-firing cells may represent penetrations of sick or dying cells.

It is also worth noting, however, that in the current experiments there is a much lower proportion of cells lacking repetitive firing abilities in WT mice than reported by Delestrée *et al*. Our criteria for classification were subtly different, potentially resulting in a higher number of cells classified as firing repetitively. From our own experience, we know that even in WT mice, cells sometimes fail to fire repetitively in response to current injection initially after being penetrated by the microelectrode, despite still displaying single action potentials. Most often, however, if tested later on in the recording they will display repetitive firing. Therefore, if a cell did not fire initially it was tested again later on in the experiment. When testing in this way and by confirming the resting membrane to be more hyperpolarized than −50 mV (using extracellular control on exiting the cells) almost all cells were found to be able to fire repetitively.

Another possible explanation for the discrepancies between studies could be due to the use of different anaesthetics. Both for the current and previous studies in the G127X mice we used Hypnorm and Midazolam anaesthesia. In contrast, in the experiments of Delestrée *et al*. a barbiturate anaesthesia was used, which is known to block PICs^42,43^. In our previous study^31^ we have shown that activation of PICs is enhanced in the G127X mouse, a finding replicated in the current experiments. This is important as increased PICs have also been observed in neonatal and embryonic motoneurones from the G93A mouse suggesting they may be a common feature of SOD1 ALS models. Therefore, it is plausible to suggest that the increase in persistent inward currents may be important for repetitive firing in SOD1 mice. The main channels mediating the Ca^2+^ PICs in motoneurones are believed to be located mainly on proximal dendrites^44–47^ where they would be activated by subthreshold excitatory input before the threshold for action potential initiation is reached at the AIS. By contrast, when using intracellular current injected directly to the soma to mimic synaptic input (as done in intracellular recording experiments), the current activates the AIS much earlier than the dendrites resulting in a delay in the activation of the dendritic Ca^2+^ channels mediating the PICs. This is then seen as the secondary range on the input-output (I-F) slope^48^. Thus, during normal physiological activation of motoneurones, the PIC activation occurs at much lower firing frequencies. For example, during intracellular current ramp injections, the simultaneous activation of afferent input to motoneurones causes activation of the secondary range at significantly lower firings frequencies than without^49^. In fact, when activated by synaptic input alone the PIC would actually be activated before repetitive firing starts and therefore the amplification of the input by PICs that underlies the secondary range would occur much earlier and therefore contribute to a steeper I-f slope in the primary range. This means that any increase in Ca^2+^ PIC would have very real consequences for repetitive firing. The exact location of the channels mediating the Na^+^ PIC is unknown but they are likely to be located at the axon initial segment as this is the highest density of channels that can mediate PICs. These are also activated at sub-spike threshold and are important for kicking off repetitive firing in motoneurones^49–51^. Therefore, both Na^+^ and Ca^2+^ PICs would have very real implications for repetitive firing in motoneurones. Given that there appears to be increases in both the Ca^2+^ and Na^+^ PIC in adult SOD1 mice, then repetitive firing would likely be more affected by anaesthesia in these animals. Our observation of an increase in input conductance is consistent with previous findings in the G93A SOD 1 mouse and the hypothesis that the increased PIC may be a mechanism to compensate for the increase in input conductance^33^.

### Functional consequences of slower maximal doublet firing

The only change with respect to multiple action potential firing was in the recovery time for action potentials initiated at the soma. The mean minimal inter-spike-interval of the 2 successive antidromic action potentials corresponded to 580 Hz in WT mice compared to 533 Hz in the G127X mice. When considering the functional implication of such a change, it is important to note that these firing frequencies are well beyond the general firing rates reported for hind limb motoneurones in awake freely moving C57BL/6 J mice^52^. During rhythmic activity, such as respiration and locomotion, however, motoneurones often initiate their active phase with high frequency doublets^53,54^. Mice hind limb motoneurones are no exception and often display doublets, or even triplets at the onset of their active phases during fictive locomotion^55^. Doublets lead to a rapid potentiation of force due to non –linear summation of the force from the two successive twitches which function to accelerate the force development at the start of movements, particularly when larger forces are required (reviewed by^56^). During fictive locomotion in mice we have observed inter-spike-intervals of between 2.6 and 11 ms, corresponding to 90–384 Hz^55^, therefore the differences in the minimum inter-spike-intervals that we observed in this study may still be considered to be outside of the normal working range during locomotion, assuming the ranges for doublets are similar in awake freely moving mice.

## Conclusion

Despite shorter AISs there was no evidence for reduced excitability within the functional working range of firing frequencies. Therefore, the conclusion from the current studies, along with our previous publications is that there is no evidence for impairments in repetitive firing in this particular ALS strain at adult presymptomatic time points. Deficits in repetitive firing should therefore not be considered a general feature of motoneurones in adult presymptomatic ALS models. It is, of course, entirely possible that this may change with disease progression in this model, therefore it will be crucial to also investigate this in the same mutant after symptom onset.

## Materials and Methods

### Ethical approval

The experimental procedures were approved by the Danish Animal Experiments Inspectorate (Permission no. 2010/561-1825 and 2013-15-2934-00879) with local approval from the Department of Experimental Medicine at Copenhagen University. All procedures were in accordance with the EU Directive 2010/63/EU for the protection of animals used for scientific purposes.

### Mice

Mice transgenically expressing G127insTGGG (G127X) mutant human SOD1(Jonsson *et al*. 2004) were backcrossed on C57BL/6 J mice for more than 25 generations in Umea, Sweden^57^. Homozygote mice from the original line 716, over-expressing 19 copies of the human SOD1 G127X gene, were then bred as homozygotes at our own institution. This model was used, as the mutant SOD1 itself lacks enzyme activity and is rapidly degraded resulting in low levels of the protein in the spinal cord^57^. This reduces the risk of over-expression artefacts associated with more commonly used mutants such as the G93A SOD1 model. The G127X strain has a relatively late clinical onset of around 250 days which is followed by a rapid progression of approximately one week. Experiments were therefore performed at ~190 days, a time point well into adulthood but prior to obvious symptom onset. Any mice starting to show symptoms were excluded. Given that the mutant SOD1 G127X has no enzymatic function and that it was necessary to breed the G127X mice as homozygotes, the best available controls were aged-matched C57BL/6 J mice, which we will refer to as wild type (WT). The numbers of mice used for each investigation are detailed in Table 1. Due to the slow breeding of our colony of the G127X, mice of both genders were used. Our previous controls have shown no effect of sex on AIS parameters in adult C57BL/6 J mice^37^.

To investigate whether differences in AIS parameter represent a strain difference existing from birth or changes that occur during adulthood in the G127X mice, we also labelled AISs in an additional 14 mice at ~22 (20–23) days of age (7 WT and 7 G127X).

### Electrophysiology experiments

In vivo electrophysiological experiments were performed on 11 G127X mice (~195 days old) and 6 age-matched C57BL/6 J mice. Experiments were performed as previously described^31,58^. Anaesthesia was induced and maintained with an intraperitoneal injection of a mixture of Hypnorm (0.315 mg/mL fentanyl-citrate and 10 mg/mL fluanisone), Midazolam (5 mg/mL), and sterile water (mixed 1:1:2) with an induction dose of this mixture of 0.15 mL/25 g body weight. Maintenance dosages of the same mixture were administered through an intraperitoneal cannula (0.05 mL every 20 minutes). All mice received a single dose of atropine (0.02 mg I.P.) at the start of the surgery. A tracheal cannula was then inserted to allow for artificial ventilation. The sciatic nerve was dissected and a hemi-laminectomy performed at vertebral levels T12-L1 allowing access to spinal levels L3-L4. Mice were then placed in a modified Narashige frame with the head secured in a head holder. The temperature was monitored using a rectal probe and maintained at 37 °C using a heat pad underneath and a heat lamp above the mouse, controlled by the output from the temperature probe. Mice were connected to a ventilator (SAR-83 CWE) and artificially ventilated at 70 breaths per minute (and a tidal volume of approximately 0.2 ml). Mice were then paralysed using the neuromuscular blocking agent Pavulon (diluted 1:10 with saline then 0.1 ml dose initially followed by 0.05 ml doses every hour). Expired carbon dioxide levels were measured with a Capstar CO_2_ analyser (IITC Life Science) and clips placed on the ear and rear foot measured the electrocardiogram (ECG) which was monitored throughout the experiment. This was used to monitor adequacy of the anaesthesia under neuromuscular blockage which was maintained by administration of the same anaesthetic (dosage as necessary for the surgical procedures). Vertebral clamps were attached on the vertebrae above and below the laminectomy to secure the spinal column in this region. A silver ball electrode was placed on the dorso-lateral surface of the spinal cord (and another down the side of the rib cage) to record the cord dorsum potentials for timing of the arrival of signals at the spinal cord (for later classification of antidromic responses). For intracellular recording, using an electronic micro-drive, a glass microelectrode (filled with 2 M Potassium acetate, resistances ~27 mΩ) was inserted into the spinal cord and antidromic field potentials from stimulation of the peripheral nerves were used as guides to locate the motoneurones. Recordings were amplified and filtered (10 kHz) using custom-made amplifiers (Copenhagen University). Finally, the intracellular signals were digitised (40 kHz) using the 1401 analogue to digital converter (Cambridge Electronic Design, UK) and recorded using the Signal software (Cambridge Electronic Design, UK).

Identification of motoneurones was made by the presence of all-or-none antidromic action potentials following stimulation of the sciatic nerve. Antidromic spikes were distinguished from synaptically driven action potentials by latency (relative to the cord dorsum potential) and arriving before any synaptic activity evoked by the stimulation was observed in the neurone. Measurements of antidromic action potentials were made from averages of successive trials (~10, averaged using Spike 2 software). Membrane potentials were measured at the time point at which averages were made and these were confirmed using extracellular potentials on exit of the cell. Analysis of spike height was performed with respect to membrane potential. Antidromic action potentials had the characteristic shape as previous defined^59^, consisting of an initial inflection in the depolarising phase caused by the presence of the initial segment (IS) spike arriving before the full somato-dendritic (SD) action potential. Averages were exported to Excel (Microsoft, US) where the first derivative was obtained. From this maximum rate of rise could be obtained for both the IS and SD components and the difference between the timing of these two points taken as the IS-SD latency^59^.

To test the recovery from inactivation, two stimuli were given in close succession and the timing between the two gradually decreased until the second antidromic action potential failed. The minimum interspike interval was then recorded.

Finally, to test the ability of the motoneurones to fire repetitively to central inputs, a ramp of increasing current was injected directly into the cell body through the microelectrode (at speeds of 5 nA/second, using DCC mode, 3 kHz on the Axoclamp amplifier) to evoke repetitive firing. Occasionally, motoneurones can fail to fire repetitively if tested immediately after penetration with the glass microelectrode. In those cases the motoneurone was tested again later in the recording. Not all features were tested in all cells in all mice and so the number of cells and mice  tested are provided in the results section. 

#### Anatomical experiments

At the end of the electrophysiological experiments mice were perfused with saline via the left ventricle, followed by a brief perfusion with 2% paraformaldehyde (in phosphate buffer, pH7.4). The spinal cords were then removed and post fixed for 1 hour in 2% paraformaldehyde. The lumbar enlargement was then cut into 50 µm thick horizontal sections on a freezing microtome and ventral sections containing motoneurones processed. Axon initial segments were labelled with a primary antibody against the voltage-gated sodium channel Nav 1.6 (Alomone 1:500) and a donkey anti-rabbit secondary antibody conjugated to Alexa Fluor 594 (Millipore 1:1000). We used Nav 1.6 as this is the main sodium channel found at the AIS responsible for action potential initiation. The Nav 1.6 antibody labelled axon initial segments (confirmed by co-labelling with Ankyrin G or Pan Nav channel antibodies) and nodes of Ranvier (confirmed by labelling of Caspr in the flanking paranodes) and pre-incubation of the antibody with its immunising peptide eliminated labelling (data not shown). Secondary-only, auto-fluorescence and cross reactivity controls produced no labelling (data not shown). Motoneurones were labelled with an antibody directed against Choline acetyletransferase (ChAT, Millipore, 1:100) and a donkey anti-goat secondary antibody conjugated to Alexa Fluor 488 (Millipore 1:1000). This labelled large ventral horn neurones (motoneurones), sympathetic preganglionic neurones in the intermediolateral column and small neurones around the central canal and intercalated nucleus, all regions known to contain neurones using acetylcholine as a neurotransmitter. By taking only horizontal sections through the ventral horn ChAT immunoreactive motoneurones could be isolated.

### AIS measurements

Confocal images of the lumbar spinal cord segments were obtained using a Zeiss LSM700 Axio Imager 2 microscope. Cells imaged were from regions of the lumbar enlargement corresponding to spinal levels L3-4 (regions containing hind limb motoneurones with axons in the sciatic nerve). Measurements of the AISs and the associated motoneurones were performed in 3 dimensions using confocal software ZEN Black (Zeiss, Germany). The following parameters of the AIS were measured in 3D: length of the AIS, distal and proximal width, distance of the proximal AIS from the cell body. Not all measurements were possible for each AIS, for example, only the AISs where both the distal and proximal ends were clearly contained within the Z-stacks were measured for length. Surface area of the neuronal cell body was measured in the x-y plane and was calculated as the area of an ellipse obtained from the minimum and maximum diameters. Identification of gamma motoneurones was based on size criterion (gamma motoneurones being less than 485 µm^2^^60^) and lack of large cholinergic boutons surrounding the gamma motoneurone soma. Due to the large size of the motoneurones and the thickness of the sections being only 50 µm, many of the motoneurone cell bodies were not entirely contained within the Z-stack, however their AISs were still included in the study if they could be identified as originating from a motoneurone (hence some gamma motoneurones may still be included in the larger sample). An extra two WT mice (not included in the table) underwent immunohistochemical labelling for Nav.1.6 but the labelling did not adequately label AISs (presumably due to too high a level of tissue fixation and the Nav1.6 antibody being very sensitive to fixation) so these mice were excluded.

### Cell counting

A further 9 mice (4 WT and 5G127X) were perfused with 4% paraformaldehyde (in phosphate buffer pH7.4) and post-fixed in the same fixative for approximately 2 hours. The lower thoracic, lumbar enlargement and upper sacral part of the spinal cord was cut into transverse 40 µm thick sections and stored in serial order. These sections were processed immunohistochemically for ChAT (same primary antibody as above) using a donkey anti-goat secondary antibody conjugated to Alexa Fluor 594. For cell counting only every 10^th^ section was used. Counting of motoneurones was carried out using an epifluorescent microscope (Axioplan 2, Zeiss, Germany). Only motoneurones with visible nuclei in their somas were counted. The rostral-caudal extent of the lumbar region of the spinal cord used for counting was defined by the end of the intermediolateral (IML) nucleus (rostral border) and the sacral parasympathetic (SPSy) nucleus (caudal border), both of which contain ChAT-immunoreactive neurones and mark the first lumbar segment and the beginning of the sacral segments, respectively. The results were plotted in serial order and aligned with respect to the last section containing IML neurones, the first section containing SPSy neurones and the central peak. Illustrative photographs were captured with a laser scanning confocal microscope (Leica LSM 700). Brightness and contrast for all illustrations was adjusted using Image J (NIH) or Photoshop (Adobe) and was applied uniformly to the entire image for illustration purposes only. All analyses, however, were performed with the raw data.

### Statistical analyses

All statistical analyses were performed using the GraphPad Prism software. D’Agostino & Pearson omnibus normality tests were used to confirm normality. For data passing this, parametric statistics (t-tests) were used. For data not passing normality tests (or analyses with a low sample number per group) non-parametric statistics (Mann-Whitney) were used. Statistical significance was accepted at the P < 0.05 level. On all graphs asterisks are used to indicate the following levels of significance: *(P < 0.05), **(P < 0.01), ***(P < 0.001). Unless indicated on the graphs no significant difference was found.

## Data Availability

The data that support the findings of this study are available on request from the corresponding author.
